# Improving the strength and toughness of macroscale double networks by exploiting Poisson’s ratio mismatch

**DOI:** 10.1038/s41598-021-92773-0

**Published:** 2021-06-24

**Authors:** Tsuyoshi Okumura, Riku Takahashi, Katsumi Hagita, Daniel R. King, Jian Ping Gong

**Affiliations:** 1 Graduate School of Life Science, Hokkaido University, Sapporo, 001-0021 Japan; 2 Department of Applied Physics, National Defense Academy, 1-10-20 Hashirimizu, Yokosuka, 239-8686 Japan; 3 Faculty of Advanced Life Science, Hokkaido University, Sapporo, 001-0021 Japan; 4 Institute for Chemical Reaction Design and Discovery (WPI-ICReDD), Hokkaido University, Sapporo, 001-0021 Japan; 5 Present Address: NTT Basic Research Laboratories and Bio-Medical Informatics Research Center, NTT Corporation, 3-1 Morinosato-Wakamiya, Atsugi, Kanagawa 243-0198 Japan

**Keywords:** Soft materials, Composites

## Abstract

We propose a new concept that utilizes the difference in Poisson's ratio between component materials as a strengthening mechanism that increases the effectiveness of the sacrificial bond toughening mechanism in macroscale double-network (Macro-DN) materials. These Macro-DN composites consist of a macroscopic skeleton imbedded within a soft elastic matrix. We varied the Poisson's ratio of the reinforcing skeleton by introducing auxetic or honeycomb functional structures that results in Poisson’s ratio mismatch between the skeleton and matrix. During uniaxial tensile experiments, high strength and toughness were achieved due to two events: (1) multiple internal bond fractures of the skeleton (like sacrificial bonds in classic DN gels) and (2) significant, biaxial deformation of the matrix imposed by the functional skeleton. The Macro-DN composite with auxetic skeleton exhibits up to 4.2 times higher stiffness and 4.4 times higher yield force than the sum of the component materials. The significant improvement in mechanical performance is correlated to the large mismatch in Poisson's ratio between component materials, and the enhancement is especially noticeable in the low-stretch regime. The strengthening mechanism reported here based on Poisson's ratio mismatch can be widely used for soft materials regardless of chemical composition and will improve the mechanical properties of elastomer and hydrogel systems.

## Introduction

The invention of double-network (DN) hydrogels demonstrated that the incorporation of a sacrificial network can result in gels that possess the toughness required for applied use. DN gels, consisting of a nanoscale interpenetrating hard and brittle “1st network” and soft and ductile “2nd network”, exhibit high Young’s modulus and high toughness despite containing 90 wt.% water^1–3^. The toughening of DN gels (known as the DN principle) occurs through the preferential fracture of “sacrificial” covalent bonds within the brittle 1st network over a wide area without causing macroscopic failure of the material^4^. Fracture of the material is delayed by the integration of the 2nd network until substantial widespread damage has occurred within the 1st network, allowing for high levels of energy dissipation. Recent advancements have shown that tough hydrogels can be fabricated not only with covalent sacrificial bonds, but also with weak, reformable non-covalent sacrificial bonds such as ionic and hydrogen bonds^5–7^. Furthermore, the DN principle not only applies to hydrogels, but also to industrial materials such as elastomers^8–11^. Implementation of the DN principle has the potential to improve the mechanical properties of wide-ranging materials systems.

Later, researchers applied the DN concept to macroscopic composites. Inspired by the DN principle, Feng et al*.* developed the first composites to explicitly demonstrate the “macroscale” DN (Macro-DN) effect, by combining a fabric mesh reinforcement with adhered VHB tape^12^. The stiff fabric mesh plays the role of the 1st network and the soft VHB tape plays the role of the 2nd network, and the composite dissipates energy by breaking the fabric mesh when force is applied, but the VHB tape enables toughness by preventing rupture of the sample. Recently, we developed Macro-DN composites made of a rigid grid skeleton imbedded in soft matrix, and these Macro-DN materials show some common features with nanoscale DN materials^13,14^. Similar to the fabric mesh example above, the roles of the stiff skeleton and soft matrix match those of the 1st and 2nd network of DN hydrogels, respectively: the skeleton dissipates energy by rupturing sacrificially, while the matrix maintains extensibility. These studies show that the DN principle can also be applied universally, regardless of size scale^15^.

Since mechanical properties of the Macro-DN composites depend much more on material selection and geometric design of the skeleton rather than molecular design, Macro-DN composites can be developed facilely by utilizing 3D printing technology. 3D printing can directly print the reinforcing phase with the desired network geometry to enable bending, rotation, or sacrificial bonds^13,16–20^. Furthermore, Macro-DN composites can incorporate functionality in this sacrificial network, by utilizing materials such as liquid or low-melting-point alloys^14,21^.

In our previous research on Macro-DN composites^13^, which utilized a rigid skeleton imbedded in a soft matrix, we focused on understanding the design parameters that control the DN principle on the macroscale. The skeleton was designed in a grid-lattice shape made up of numerous sections, with stiff crossbars delineating each section. The stiff crossbars prevented transverse deformation of the composites when stretched. Rupture of rigid interconnects in multiple sections occurs when the strength ratio between the reinforcing skeleton and matrix approaches 1, enabling high fracture force and large extensibility to show the maximum toughness. In such composites, the matrix of the composite carries almost no stress before breaking the rigid lattice, and the yield force and stiffness of the Macro-DN composite are comparable to that of the individual skeleton. The role of the soft matrix was to maintain global integrity of the composite after breaking of the rigid interconnects, and local deformation only occurs in the regions that have fractured. Furthermore, the maximum strength of the composite was limited to the fracture strength of the skeleton in the low stretch region, and to the matrix at ultimate fracture. The combination of these two points limits the increase in toughness to approximately a factor of two, when compared to a neat matrix sample.

In order to achieve higher strength and toughness in Macro-DN composites, it is necessary to introduce a unique macroscopic mechanism in which the deformation of the skeleton and the matrix are strongly coupled even before the rupture of the skeleton. In this case, the skeleton not only dissipates energy through fracture but also acts to apply stress on the matrix to increase the strength of the composite in a synergistic manner. It is known that preferential design of a reinforcing phase in composites with interpenetrating phase structures enable additional plastic deformation, resulting in increased strength and toughness^22–24^. This effect has been demonstrated on both the macro- as well as the microscale^25^. Recently, it has been shown that 2D re-entrant honeycomb reinforced structures that exhibit auxetic characteristics under compression improve mechanical performance such as impact resistance when modulus mismatch exists between the two phases^17–19,26,27^. Here, our interest is in soft composite materials that can exhibit high strain at break with ductile characteristics under tensile deformation, along with high stress. To realize this effect, we present a strategy to design skeletons that can exhibit transverse deformations under uniaxial tensile strain prior to rupture as sacrificial bonds. We design sacrificial skeleton networks that can increase or decrease their planar area with deformation, which induces size mismatch with the soft matrix that intends to maintain an almost constant volume during deformation. The correlation between the longitudinal and lateral deformations of materials is characterized by Poisson’s ratio (μ), defined as the negative ratio of lateral contraction strain (ε_x_) to the longitudinal extension strain (ε_y_) of a material (or structure). The Poisson’s ratio of isovolumetric materials is μ $=$ 0.5. In contrast, planar honeycomb structures have a Poisson’s ratio larger than 0.5, while planar auxetic structures that expand in the lateral direction when being stretched have a negative Poisson’s ratio^28–30^. Both honeycomb and auxetic structures provide simple methods to tune the mechanical coupling between the rigid skeleton and soft matrix in Macro-DN composites, which can be characterized by the difference in the Poisson’s ratio, Δμ = μ_skeleton_–μ_matrix_.

In this work, we fabricated three categories of two-dimensional skeleton structures: auxetic, offset rectangle, and honeycomb, and investigated the mechanical properties of the macroscale planar composites by uniaxial tensile testing. The mismatch in Poisson’s ratio exerts biaxial stress on the soft matrix before the skeleton ruptures, and the mechanical behaviors and the rupture processes of the composites were analyzed. Taking advantage of the planar structure, we also performed real-time birefringence visualization of the stress distribution during uniaxial tension. The stiffness, fracture force, and work of extension were characterized for each composite as a function of the Macro-DN Poisson’s ratio mismatch, Δμ. We see that when the magnitude of Poisson’s ratio mismatch is high, regardless of whether it is negative or positive, the impact of reinforcement significantly increases. Furthermore, we show that these Macro-DN composites exhibit enhanced toughness due to improved matrix deformation by large Poison’s ratio mismatch with the skeleton, rather than just through sacrificial bonds. This mechanism greatly increases low stretch mechanical properties compared to the component materials, resulting in a ~ 320% increase in stiffness, a ~ 340% increase in yield force, and a ~ 200% increase in work of extension for a stretch ratio up to half of the fracture stretch ratio. From these results we propose a new strengthening mechanism for Macro-DN composites via Poisson’s ratio mismatch.

## Results

### Design of Macro-DN composites with Poisson’s ratio mismatch

**Figure 1:**
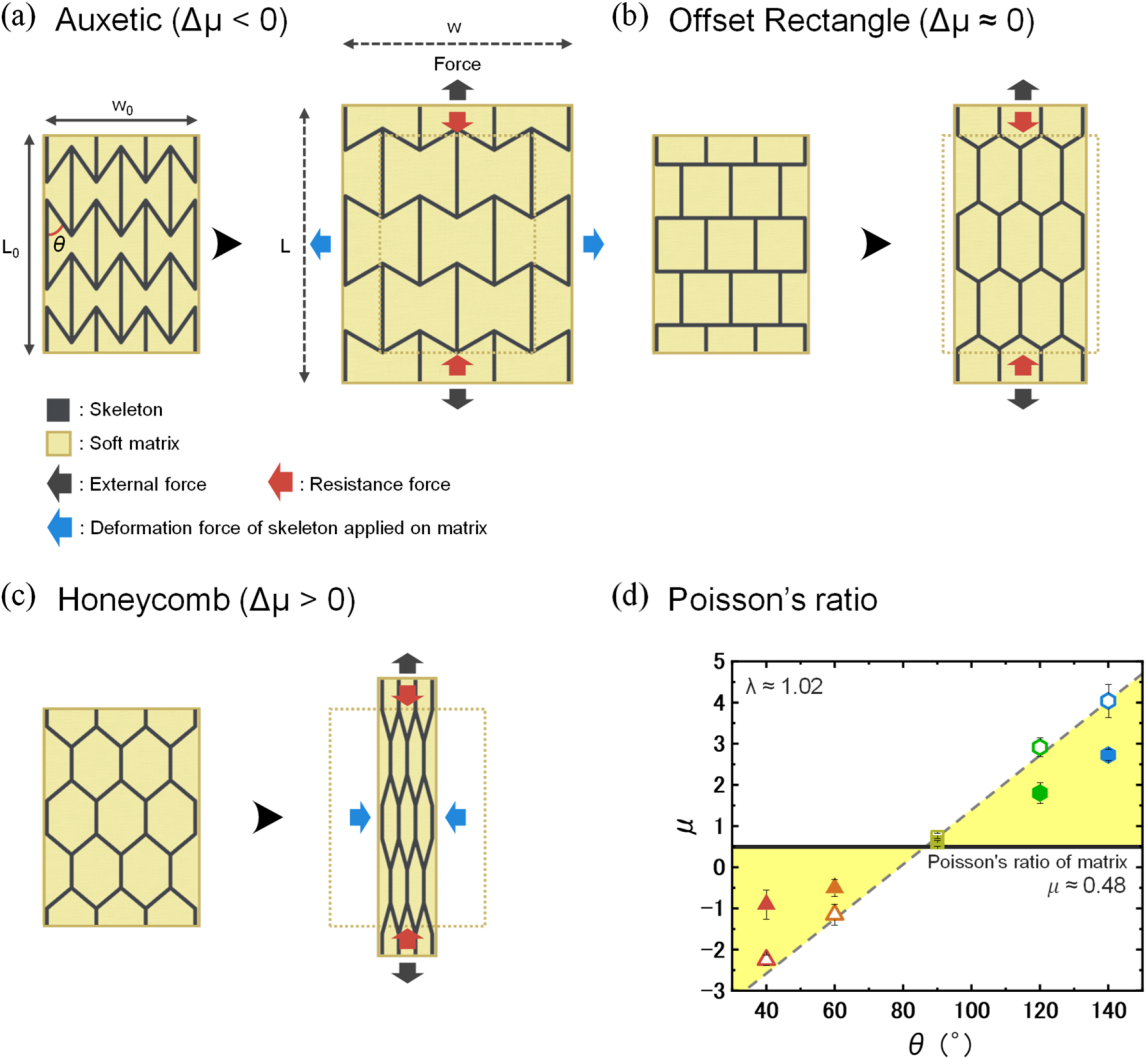
Hypothesis of the mechanism by which Poisson’s ratio mismatch between components influences the deformation process of Macro-DN composites. Predicted deformation and force balance of (**a**) an auxetic composite, (**b**) an offset rectangular composite, and (**c**) a honeycomb composite. Here, each skeleton mesh shows how it deforms differently in response to tensile force, due to their varying planar Poisson’s ratio, μ. The internal angle and defined lengths for measuring Poisson’s ratio are shown in (**a**). The auxetic skeleton causes the matrix to undergo biaxial extension, while the honeycomb composite causes the matrix to experience compressive forces. (**d**) Poisson’s ratio of matrix (solid black line), skeleton (open symbols), and composite (filled symbols) as a function of skeleton angle, θ. The exact design of these skeletons is shown in Figure S1. The highlighted yellow areas represent regions where Poisson’s ratio mismatch (∆μ) occurs between skeleton and matrix. The measured Poisson’s ratio of composites falls between the measure value of the skeleton and matrix. The dashed line is a linear regression for the relation between angle, θ, and Poisson’s ratio of skeleton, μ_skeleton_.

As model materials, the rigid skeleton is made from a polyurethane/polyacrylate (PU/PA) copolymer resin that is formed by 3D printing, and the soft matrix is a commercial elastic silicone rubber. The mechanical properties of these two materials are significantly contrasting, with the PU/PA resin possessing a modulus more than three orders of magnitude greater than the silicone (Table S1). The composites are designed based on three types of geometries: auxetic, offset rectangle, and honeycomb skeletons (Fig. 1). Specific details can be seen in Supporting Information 2 and Figure S1-S2. First, we will analyze the neat skeletons independently, schematically shown in black. Poisson’s ratio (μ) is defined as the negative ratio of transverse contraction strain (ε_x_) to the longitudinal extension strain (ε_y_) of a material as follows:

1 $${\varepsilon }_{x}=\frac{(w-{w}_{0})}{{w}_{0}},\,{\varepsilon }_{y}=\frac{(L-{L}_{0})}{{L}_{0}},\, \mathrm{\mu }=-\frac{{\varepsilon }_{x}}{{\varepsilon }_{y}}$$

where L and L_0_ represent the current length and initial length along the stretching direction, and w and w_0_ represent the current width and initial width perpendicular to the stretching direction, respectively (Fig. 1a). We systematically change the planar Poisson’s ratio of the skeleton by changing the interior angle θ of the mesh. Specifically, an auxetic mesh (θ < 90°) has a negative Poisson's ratio, showing expansion in the dimensions normal to the stretching direction, as shown in the representation in Fig. 1a. An offset rectangle mesh (θ = 90°) has a Poisson's ratio near 0.5, as shown in Fig. 1b. On the other hand, a honeycomb mesh (θ > 90°) has a Poisson's ratio much larger than 0.5, showing large contraction in the dimensions normal to the stretching direction, as shown in Fig. 1c. Figure 1d shows the measured Poisson's ratio of the matrix (black solid line) and each skeleton with different internal angle (open symbols). These values are calculated by using images of the samples taken at a stretch ratio, λ = $\frac{L}{{L}_{0}}$  = 1.02 during tensile testing. An example of how planar Poisson’s ratio was measured is shown in Figure S3. The pristine matrix has a Poisson's ratio of μ_matrix_ = 0.48. In the case of the neat skeletons, the Poisson's ratio, μ_skeleton_, could be controlled from below -2 to 4 as the internal angle, θ, increases. The relation between μ_skeleton_ and θ could be represented by a linear regression (dashed line) in Fig. 1d.

By introducing these functional skeletons with different internal angles into a nearly incompressible soft matrix (shown in gold in Fig. 1a–c), we can produce two regions where mismatch of Poisson's ratio between skeleton and matrix, ∆μ = μ_skeleton_—μ_matrix_, changes from negative to positive (Fig. 1d, yellow regions). We aim to clarify the effect of this mismatch on the mechanical behavior of the Macro-DN composites. Depending on the functional structure, the skeleton will apply a different biaxial stress (e.g. compression or extension) on the matrix during deformation. Figure 1a shows a schematic of an auxetic mesh (μ_skeleton_ < 0) embedded within an elastic, nearly incompressible matrix. Upon stretching by an external force, the 2D auxetic mesh expands in the lateral dimension while the matrix intends to contract in lateral direction. If the mesh is stiffer than the matrix, this negative mismatch in Poisson's ratio (∆μ < 0) will cause the matrix to undergo biaxial extension, denoted by the blue arrows. Due to the near-incompressibility of the matrix, it will contract in the thickness direction. For the offset rectangular structure (μ_skeleton_ ≈ 0.5), the Poisson’s ratio mismatch is very small (∆μ ≈ 0), and both skeleton and matrix deform similarly with negligible force transfer between the two components (Fig. 1b). Figure 1c shows a schematic of a honeycomb structure (μ_skeleton_ > 0.5) embedded within an elastic matrix. In this case, the positive Poisson’s ratio mismatch (∆μ > 0) will apply a lateral compression to the matrix at stretching, which causes the matrix to expand in the thickness direction to compensate for this biaxial compression. In both cases, the matrix is undergoing additional deformation that would not occur when the Poisson’s ratio of the skeleton and matrix are matched. The Poisson's ratio of the resulting composites as a function of the internal angle of the skeleton are shown as solid symbols in Fig. 1d. Poisson’s ratio of the composites closely matches the numerical average of the two components,$\frac{{\mu }_{skeleton}+{\mu }_{matrix}}{2}$, due to the similarity in stiffness of the two phases (Figure S4).

### Tensile behavior of composites with varying mismatch in Poisson’s ratio

**Figure 2:**
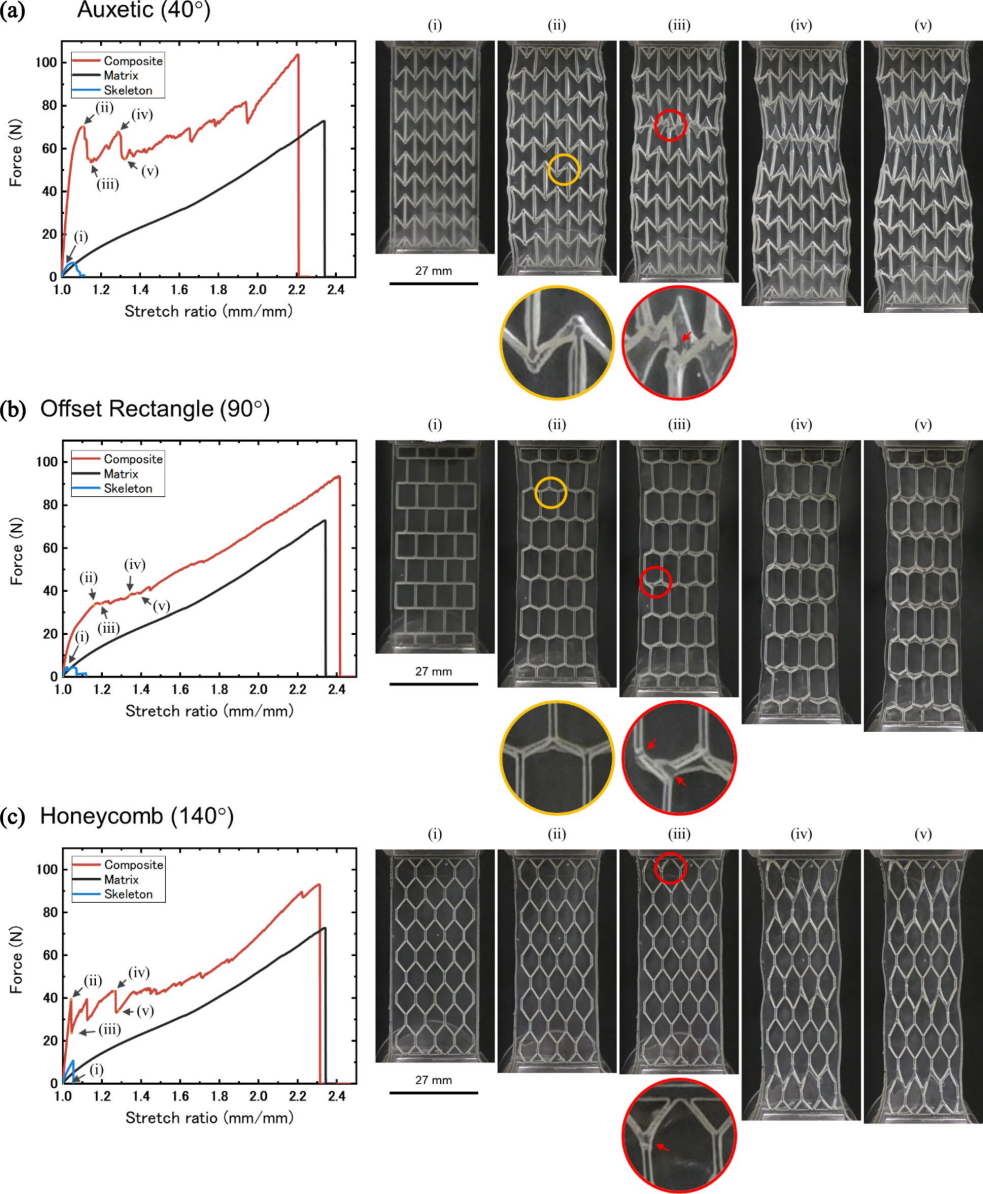
Uniaxial tensile behavior and internal fracture of the Macro-DN composites with skeletons of varying structure. Force-stretch curves of a neat skeleton (blue), pristine silicone rubber (black), and their composite (red). Yellow circles and red circles show where the delamination between the component materials and fracture of the skeleton occurred, respectively. (**a**) A composite with auxetic skeleton. (i–v) Images correspond to the labels in (**a**). (**b**) A composite with offset rectangle skeleton. (i–v) Images correspond to the labels in (**b**). (**c**) A composite with honeycomb skeleton. (i–v) Images correspond to the labels in (**c**).

To demonstrate the impact of the functional skeleton designs described in Sect. 2.1, we first measure and compare the mechanical properties of the soft matrix, representative functional skeletons, and the resulting composite structures by tensile testing. Representative samples with internal angles of 40° (auxetic, −∆μ), 90° (offset rectangle, ∆μ ≈ 0), and 140° (honeycomb, +∆μ), are shown in Fig. 2a–c, respectively. From the resulting force versus stretch ratio curves, we see that the neat silicone rubber is elastic, fracturing at a stretch ratio of approximately 2.3. By comparison, the skeletons are much more brittle, fracturing at a stretch ratio of only ~ 1.1. In our previous research, we showed that when the fracture force of the 1st network matched that of the 2nd network, the mechanical properties of DN materials are optimized. However, in this system, the skeleton fracture forces are much lower than the matrix fracture force, and the skeleton stiffnesses are similar to that of the matrix, because the incorporated joints allow the skeleton to bend easily, despite the skeleton consisting of a much higher modulus material.

All composites had far better mechanical properties than the component materials. Surprisingly in the low stretch ratio region, the composites exhibited much higher force and stiffness than the neat skeleton. In previous Macro-DN composites, the initial peak force represented the yielding force of the composite, and this value matched the fracture force of the skeleton. In the designs utilized here with compliant skeletons however, the yielding force greatly exceeded the fracture force of the skeleton. The force versus stretch ratio curves along with corresponding pictures will be described in order.

First, we will examine the results of the auxetic (40°), −∆μ composite in Fig. 2a. Images of the sample at corresponding stretch ratios can be seen in Fig. 2a(i–v). Upon stretching, a yield point is reached at a stretch ratio of 1.1, corresponding with the expansion of the skeleton (Fig. 2a(ii)). At this time, since the interfacial bonding strength between the component materials is small, local delamination is observed. However, this does not result in sample failure because mechanical interlocking prevents large-scale delamination from occurring between the component materials. The yielding force is 68.5 ± 1.50 N, greatly exceeding the sum of the forces of the neat matrix and skeleton at that stretch ratio, demonstrating a synergistic increase in strength from the composite structure. After reaching the yield point the force of the composite dropped, representing fracture of the skeleton (Fig. 2a(iii)). Load is then transferred from the skeleton to the matrix. With additional stretch, the sample became increasingly distorted, demonstrating high local deformation. With increasing stretch, the skeleton began to fracture preferentially numerous times, enabling high toughness (Fig. 2a(iv, v)). These fracture processes show that the skeleton acts as a 1st network and dissipates energy, and the matrix acts as a 2nd network and supports the bulk of the load on the composite, in agreement with our previous work and double network theory. Finally, in the high stretch ratio region, the force of the composite exceeded that of the pristine matrix, even though the skeleton contained many fracture sites. The final fracture force was 98.53 ± 4.37 N, greatly exceeding the sum of the matrix and skeleton.

Next, we examine the offset rectangle (90°), ∆μ ≈ 0 composite. Images of the sample at corresponding stretch ratios can be seen in Fig. 2b. While the yield force is again much higher than the skeleton fracture force, compared to the auxetic composite the yield force is approximately half (32.18 ± 2.05 N versus 68.52 ± 1.50 N), and occurs at a larger stretch ratio (~ 1.2). During deformation, we do not see a pronounced change in lateral geometry, in contrast to the auxetic composite (Fig. 2b(ii)). Interestingly, in the ductile region, a jig-saw shaped force versus stretch curve was not observed, as has been previously seen in Macro-DN composites and in the auxetic composite. This demonstrates less load is supported solely by the skeleton, and the fracture process results in less transfer of force between the skeleton and matrix. However, as in the case of the auxetic composite, due to the deformation of the skeleton, slight local delamination is observed between the component materials. After internal fracture (Fig. 2b(iii)) of the sacrificial bonds, global fracture of the sample occurs at ~ 90 N, greater than the sum of the components but less than the fracture force seen in the auxetic composite.

Finally, Fig. 2c shows the force versus stretch ratio curve of a honeycomb (140°), +∆μ composite along with images of the sample at corresponding stretch ratios. The yield force is reached quickly at only a stretch ratio of 1.05 and achieves a force (38.4 ± 1.85 N) that exceeds the offset rectangle composite but is less than the auxetic composite. After the yield point, rupture of the skeleton occurs (Fig. 2c(iii)), and the force drops. Since the yield force is reached at a low stretch ratio, the honeycomb composite does not show local delamination between the component materials as is seen in the auxetic and the offset rectangle composites. Increasing stretch ratio causes further deformation and sacrificial of the skeleton (Fig. 2c(ii–v)), before final fracture occurs at a force exceeding the sum of the fracture forces of the skeleton and matrix. The global fracture force follows the general trend, greater than the offset rectangle design, and less than the auxetic composite.

**Figure 3:**
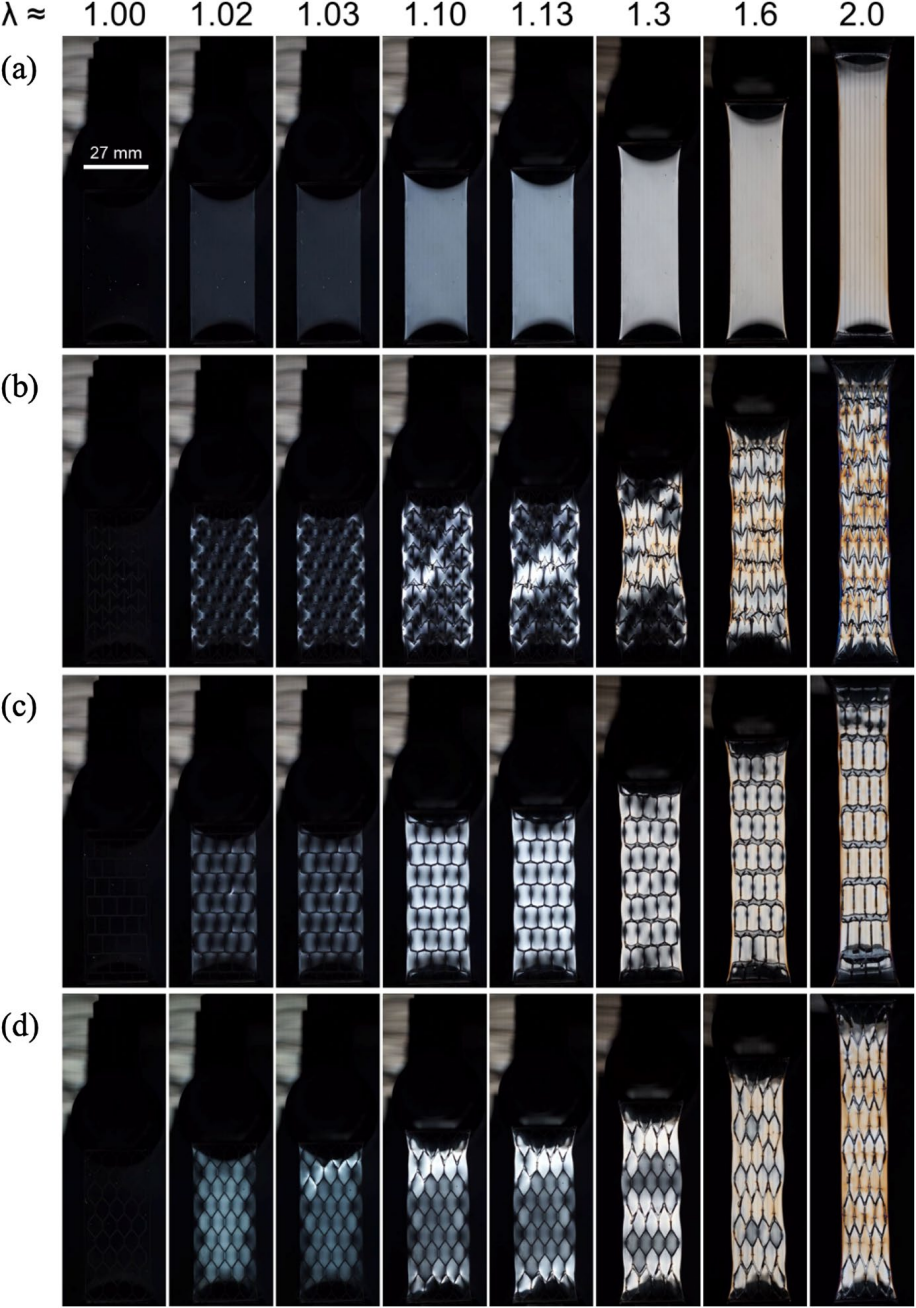
Circular polarized images of the Macro-DN composites under uniaxial tension. (**a**) Neat matrix. (**b**) Auxetic skeleton (Δμ = − 2.73). (**c**) Offset rectangle skeleton (Δμ = 0.26). (**d**) Honeycomb skeleton (Δμ = 3.56). The completely dark images at λ = 1 demonstrates the stress-free state of the composites prior to stretching, while the increase in coloring at λ > 1 reveals the internal stress distribution of the samples.

The circular polarized images of the composites and a neat matrix sample during the tensile tests were taken (Fig. 3, Video S1). These images visualize the magnitude of the stress distribution during deformation of the composites. As seen from the neat matrix sample (Fig. 3a), in the unstretched state the sample has no molecular orientation and therefore appears completely dark. With stretching, we see a continual homogenous increase in the intensity of the coloring, turning from white to orange with the increase of stretch. In the case of the auxetic, −∆μ composite, regions in close proximity to the angled joints that bend with deformation initially become bright before the matrix that lies in the center of a unit cell (Fig. 3b, λ = 1.02, 1.03). In the high stretch ratio region, even after rupture occurs within the skeleton, the stress is concentrated near the ruptured joints at a level that is higher than the stress in the pristine matrix at the same stretch (Fig. 3b, λ = 1.6, 2.0). For the offset rectangle composite with little Poisson’s ratio mismatch, the magnitude of deformation as judged by the coloring matches closely to that of the pristine matrix (Fig. 3c). Because Δμ ≈ 0, the skeleton does not impose additional stress on the matrix, and the magnitude of deformation in the matrix with increasing stretch is similar to the neat matrix. Finally, Fig. 3d contains the images of the honeycomb, + Δμ composite. In the low stretch region, λ = 1.01, the initial deformation appears in the center of the skeleton unit cell, due to the compression imposed by the skeleton. This result is significantly different from what was seen in the auxetic, − Δμ composite that undergoes local biaxial extension. Even at high stretch this observation continues (λ = 2), with brighter coloring occurring in the bulk of the unit cell rather than at the joints. Analysis via circular optical polarization confirms the stress exchange mechanism due to Poisson’s ratio mismatch during the deformation in Macro-DN composites with functional structures.

An interesting observation from the images in Fig. 3 and Video S1 is that all samples exhibit deformation homogenously throughout the length of the composite at low stretch. This type of deformation process occurs because the stiffness of the functional skeletons introduced here allow for global deformation at low stretch, and the skeleton and matrix are closely matched in stiffness. By comparison, in our previous Macro-DN design that was based on a rigid square-lattice design^13^, the skeleton was much stiffer than the matrix, resulting in an individual region undergoing significant deformation prior to the rupture of another region. With a very stiff skeleton, at small global stretch only a small portion of the sample (~ 10%) can deform. Due to Poisson’s ratio mismatch and comparable stiffness, we observe a yield force and stiffness significantly greater than the neat skeleton, in contrast to the previous design where both yield force and stiffness match that of the skeleton.

### Comparison of mechanical properties in the low stretch ratio region

The primary way in which the mechanical response introduced here differs from previous research is the significant increase in yield force, compared to the neat components at similar stretch. To investigate the origins of this response, we will focus our study to the lower half of the stretch region, up to λ = 1.65. We investigated the mechanical properties of the composite based on systematic modification to the geometry of the skeleton. The interior angle, θ, of the skeleton was varied between 40° and 140°, resulting in five types of skeletons exhibiting either auxetic (− Δμ), offset rectangle (Δμ ≈ 0), or honeycomb (+ Δμ) response (Figure S1c). The mechanical properties were investigated by uniaxial tensile tests in the same way as Sect. 2.2.

First, we investigated the difference in mechanical properties of the neat components, with the results shown in Table 1. All skeletons exhibit similar stiffnesses, regardless of Poisson’s ratio. Yield force and stretch ratio are also similar, with the exception of the 140° skeleton, that has a higher yield force, likely due to the high degree of alignment from the high internal angle. In Table 1 we can see that both skeleton and matrix exhibited similar stiffnesses despite having neat material moduli that differ by three orders of magnitude (Table S1). One significant difference between the two components is that the matrix, as an elastic dissipator, has a much higher work of fracture.

**Table 1:** Mechanical properties of the neat skeletons and pristine matrix by uniaxial tensile testing.

Component	Structure	Angle, θ (°)	Poisson’s ratio, μ	Initial stiffness, κ (kN/m)	Yield force, F_y_ (N)	Yield stretch ratio, λ_y_	Work of fracture, W (mJ)
Skeleton	Auxetic	40	− 2.25 ± 0.13	3.48 ± 0.06	6.41 ± 0.14	1.058 ± 0.003	23.77 ± 2.44
Skeleton	Auxetic	60	− 1.15 ± 0.25	3.40 ± 0.25	5.40 ± 0.08	1.047 ± 0.005	14.52 ± 2.62
Skeleton	Offset Rectangle	90	0.74 ± 0.09	4.84 ± 0.24	4.80 ± 0.02	1.021 ± 0.003	23.25 ± 0.35
Skeleton	Honeycomb	120	2.91 ± 0.23	3.50 ± 0.24	5.87 ± 0.55	1.054 ± 0.016	14.47 ± 5.59
Skeleton	Honeycomb	140	4.04 ± 0.40	3.95 ± 0.18	12.47 ± 2.61	1.066 ± 0.012	28.64 ± 5.59
Matrix	–	–	0.48 ± 0.01	1.69 ± 0.02	–	–	2885 ± 312

**Figure 4:**
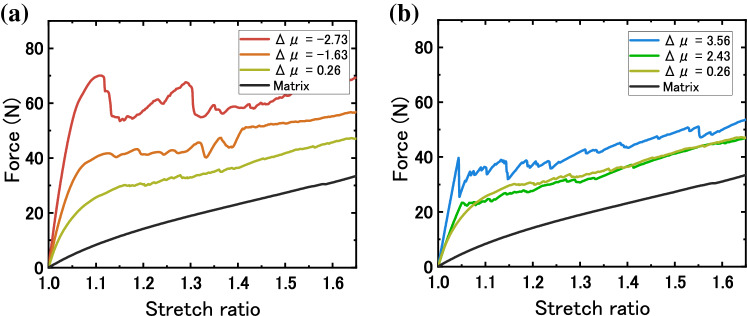
Mechanical response of composites in the low stretch region (λ = 1.65). (**a**) Force versus stretch ratio curves for composites with internal angle equal to or less than 90°. (**b**) Force versus stretch ratio curves for composites with internal angle equal to or greater than 90°. As internal angle deviates from 90°, initial stiffness, yield force, and work of extension increases.

Next, we examine the force-stretch curves of the composite and the matrix, up to half of the maximum stretch ratio, λ = 1.65. Figure 4a includes the samples with θ $\le$ 90°, and Fig. 4b includes the samples with θ ≥ 90°. In Fig. 4a, we see that as the Δμ decreases to more negative values, yield force increases. The stretch ratio at which yielding occurs for the auxetic composites is much greater than what is seen for the neat skeletons (Table 1). In Fig. 4b, we see the mirror effect: as Δμ increases to more positive values, yield force also increases, but less prominently in comparison with the case of negative Δμ. For the honeycomb samples, the yield force occurs at a stretch ratio similar to that of the neat skeletons. The offset rectangle skeleton with an internal angle of 90° shows a Poisson’s ratio closes to that of the neat matrix and shows an increase in yield force due to sacrificial fracture of the skeleton. In summary, as the internal angle of the skeleton deviates from 90° and the absolute magnitude of Δμ increases, we see a further increase in force as a function of stress, but the Poisson’s ratio mismatch effect is much prominent in the case of negative Δμ.

**Figure 5:**
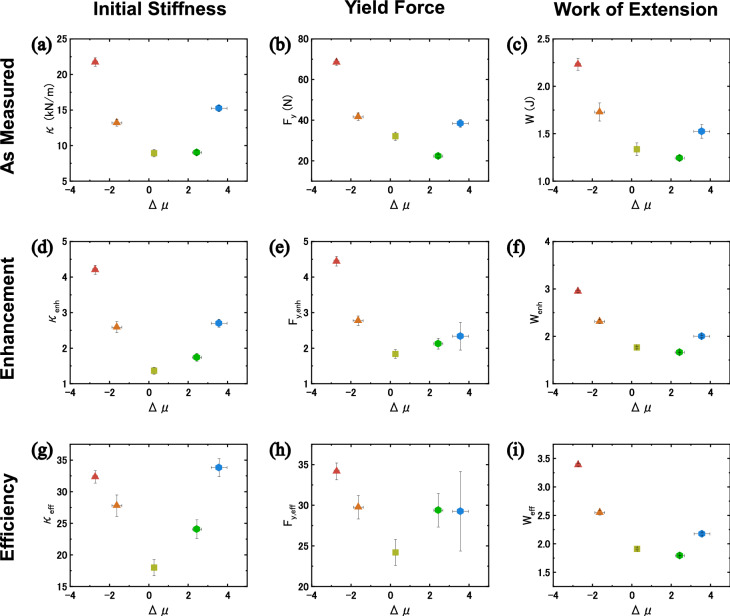
Effect of Macro-DN Poisson’s ratio mismatch, Δμ, on the measured value, the enhancement ratio, and the efficiency ratio of the composite mechanical properties up to λ = 1.65. Relationship between Δμ and measured (**a**) stiffness, (**b**) yield force, and (**c**) work of extension. Enhancement ratio versus Δμ for (**d**) stiffness, (**e**) yield force, and (**f**) work of extension. Stiffness, yield force and work of extension are normalized by the sum of the component materials. Efficiency ratio versus Δμ for (**g**) stiffness, (**h**) yield force, and (**i**) work of extension. Stiffness and yield force are normalized by the volume fraction of the skeleton, and work of extension is normalized by the volume fraction of the matrix. Error bars for all plots represent the standard deviation from n > 3 samples.

To more clearly see the effect that skeleton geometry has on the composites, we plotted the initial stiffness (Fig. 5a), yield force (Fig. 5b), and work of extension (Fig. 5c) of the composites as a function of Δμ. Work of extension was calculated as the area under the force versus stretch ratio curve, $W={L}_{0}{\int }_{\lambda =1}^{\lambda =1.65}Fd\lambda$. While all composites show increased performance compared to the neat materials, we see that modifying the structure of the skeleton to possess Δμ with a large magnitude, either in the positive or negative direction, increases the mechanical performance of Macro-DN structures compared to the Δμ ≈ 0 design.

To better understand the improvement in mechanical properties of the composites we calculate the enhancement ratio (Fig. 5d–f). The enhancement ratio for initial stiffness (κ_enh_), yield force (F_y,enh_), and work of extension (W_enh_) are calculated as follows:

2a $$\kappa _{{enh}} = \frac{{\kappa _{{composite}} }}{{\kappa _{{skeleton}} + ~\kappa _{{matrix}} }}$$

2b $$F_{{y,enh}} = \frac{{F_{{y,~composite}} }}{{F_{{y,skeleton}} + F_{{matrix}} \left( {\lambda _{{y,composite}} } \right)}}$$

2c $$W_{{enh}} = \frac{{W_{{composite}} }}{{W_{{skeleton}} + W_{{matrix}} }}$$

where the subscripts refer to the property of the specific component. The enhancement ratio describes the increase in a given mechanical response compared to the simple addition of each component: an enhancement ratio greater than 1 shows a synergistic increase in mechanical properties. In Fig. 5d, the initial stiffness of the most strongly auxetic and honeycomb reinforced composites was enhanced by up to 4.2 × and 2.7 ×, respectively, while the offset rectangle was enhanced by only 1.4 ×. The larger the magnitude of Δμ, the higher the initial stiffness of the composite. To calculate the enhancement of yield force, we estimated the matrix contribution to yield force by using the force in the matrix at the λ where the composite exhibits yielding, because the matrix does not exhibit a yield point independently. For yield force, the most highly auxetic skeleton reinforced composite showed an enhancement of up to 4.4 ×, and this value decreases as we approach Δμ ≈ 0 (Fig. 5e), which exhibits an enhancement of 1.8 ×. However, as Δμ increases we see a slight increase in enhancement, with the most elongated honeycomb composite having a yield force enhancement of 2.3 ×. Because the incorporation of functional skeletons results in fracture forces that are greater than the fracture force of the combined neat components, we have developed a method to overcome a limitation of our previous macroscale design.

To understand why the response was stronger in auxetic than honeycomb composites, we compared the stretch ratio when yielding occurs for the neat skeleton and the composite (Figure S5). We see that the yielding stretch ratio is higher in auxetic and offset rectangle composites than in their neat skeletons but is lower in the honeycomb composite than the neat skeleton. From this finding, we believe that in the honeycomb composites, the matrix inhibits the free deformation of the skeleton during extension and resulting in greater stress within the skeleton of the composite than for the neat skeleton, resulting in early rupture of the reinforcing phase. While the enhancement of yield force for the honeycomb composites is not as pronounced, these composites still exhibit high toughness. The work of extension shows the same tendency as the initial stiffness, and the auxetic and honeycomb composite work of extension can be enhanced by up to 2.9 × and 2.0 × (Fig. 5f), respectively, when compared to the neat matrix. We can clearly see that as the *magnitude* of Δμ increases in *either* the positive or negative direction, the better the mechanical properties of the composite. Interestingly, this result differs from the previous results of auxetic co-continuous composites prepared by Wang and coworkers, which exhibited a maximum in energy dissipation close to μ ≈ 0.^17^.

When designing the functional skeletons, we aimed to keep the number of sacrificial bonds and the cross-section of these bonds constant, so that the stiffness and influence of sacrificial rupture could be compared among all skeletons. By maintaining this constant geometry, the volume fraction of the skeletons within the composites (φ_skeleton_) were different, as shown in Table S2. The skeleton volume fraction of the offset rectangle structure and honeycomb structures were approximately 8%, while the most highly auxetic structure reached 13%. Taking the volume fraction into account, we estimated the efficiency ratio (Fig. 5g–i) for the initial stiffness (κ_eff_), yield force (F_y,eff_), and work of extension (W_eff_). These terms are calculated by:

3a $$\kappa _{{eff}} = \frac{{\kappa _{{enh}} }}{{\varphi _{{skeleton}} }}$$

3b $$F_{{y, eff}} = \frac{{F_{{y,enh}} }}{{\varphi _{{skeleton}} }}$$

3c $$W_{{eff}} = \frac{{{\text{W}}_{{enh}} }}{{\varphi _{{matrix}} }}$$

where φ_skeleton_ and φ_matrix_ represent volume fraction of neat skeleton and pristine matrix, respectively. Energy dissipation takes places through two methods: (1) deformation of the elastic matrix by the skeleton, and (2) rupture of the sacrificial bonds within the skeleton. Since each skeleton regardless of design has the same number of sacrificial bonds, we assume that the work of extension is dependent on the volume fraction of the matrix. In Fig. 5g, we see the efficiency results match those of enhancement ratio: as Δμ deviates from 0, stiffening efficiency increases. Despite having different volume fractions of skeleton, the auxetic, −Δμ composites and the honeycomb, +Δμ composites exhibit similarly high stiffening efficiencies. Regarding the yield force, Fig. 5h, we again see the highest efficiency in the most highly auxetic composite, and the lowest value in the offset rectangle composite. The honeycomb composites also outperform the offset rectangle composite. Finally, Fig. 5i shows the work of extension versus Poisson’s ratio. The auxetic structure shows the highest efficiency, as it can dissipate the most energy, even though it possesses less matrix to deform than the other composites. The honeycomb structure with the largest Poisson’s ratio mismatch exceeded the offset rectangle work of extension efficiency but was lower than the auxetic design. In conclusion, we consider that the mismatch of Poisson’s ratio between skeleton and matrix plays an important role for increasing the mechanical properties of Macro-DN designs, even when taking the loading fraction into account.

### Investigating the impact of skeleton design in the elastic (no fracture) region

Traditionally the DN principle works to increase toughness by the rupture of sacrificial bonds, which occurs beyond the yield point. The incorporation of functional sacrificial networks results in significant improvement in strength (stiffness and maximum force) and toughness (work of extension), even in the stretch regime where the response is primarily elastic, and the network has not yet fractured. We studied the mechanical response in the stretch region prior to fracture to understand the role of functional structures on the deformation process.

**Figure 6:**
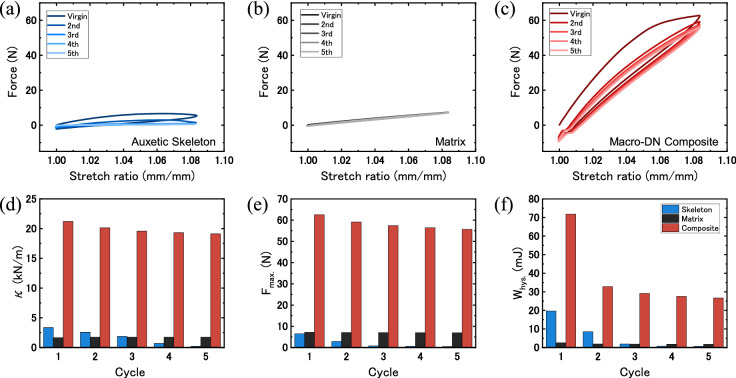
Cyclic tensile behavior of the auxetic, −Δμ Macro-DN composite. Force-stretch curves of (**a**) a neat auxetic (40°) skeleton, (**b**) the neat matrix (silicone rubber), and (**c**) the resulting Macro-DN composite. Comparisons of (**d**) stiffness, (**e**) maximum force, and (**f**) energy dissipation up to λ = 1.083, for the skeleton (blue), matrix (black), and composite (red) at different cycle numbers.

In order to show the impact that the Macro-DN design has even without skeleton fracture, cyclic tensile testing was conducted up to a strain of ~ 8% (λ = 1.083), where the skeleton does not yet fracture in the composite (λ < λ_y_). As an example, we used the auxetic (40°), −Δμ composite, which exhibited the greatest enhancement in mechanical response, according to Sect. 2.3. First, the neat skeleton was tested (Fig. 6a). Stress concentrations in the joints of the skeleton during the first stretching resulted in partial skeleton rupture at only a stretch ratio of 1.06. During subsequent stretching cycles, the mechanical properties of the skeleton continued to decrease. It should be noted that since the skeleton was plastically deformed by stretching, a slight negative force was observed when the stretch ratio returned to its original position. Next, the experiment was repeated for the pristine matrix (Fig. 6b). The force generated by this test was very low but did not change upon with cycling, because the silicone elastomer is highly elastic. On the other hand, as seen in Fig. 6c, in the composite the mechanical properties improved significantly compared to the neat components, exhibiting increased stiffness and maximum force. No fracture occurred in the skeleton within this stretch ratio region. The composite shows a compressive force after the first stretching cycle, and a decrease in maximum force after the first cycle. These results can be attributed to two effects. First, as mentioned before, during stretching the skeleton exhibits some plastic deformation. Upon unloading, the shape of the skeleton has slightly changed, resulting in compression when returning to the original position. Second, we can see from the videos of the stretching tests (Video S2) that the matrix is able to debond from the skeleton during stretching, and this only occurs in the first stretching cycle. Despite these effects, even after repeated stretching the mechanical properties of the composite are superior to the component materials.

A comparison of the stiffness, maximum force, and energy dissipation (difference in work of extension during loading and unloading) can be seen in Fig. 6d–f, respectively, for each component and the composite over five cycles. Since the skeleton begins to break, each subsequent step causes the skeleton to decrease in stiffness and force. The pristine matrix and composite, in contrast, exhibit almost no change within the first five cycles. The energy dissipation exhibits more obvious changes. The energy dissipation was estimated from the hysteresis area, W_hys_, by:

4 $${W}_{hys}={L}_{0}{\int }_{\lambda =1}^{\lambda =1.083}({F}_{load}-{F}_{unload})d\lambda$$

where F_load_ and F_unload_ are the force during loading and unloading, respectively.

The skeleton and matrix show the same trends as before; the energy dissipation of the neat skeleton decreases with increasing cycle, while the matrix exhibits consistent but low energy dissipation. For the composite sample from the second cycle, the energy dissipation decreased by half, but the skeleton did not fracture. This decrease in hysteresis occurs due to the debonding of the matrix from the skeleton, resulting in the loss of an energy dissipation source. However, despite some degree of delamination occurring, there was no catastrophic failure of the sample, and continued cycling can occur without further decrease in performance. This result shows the importance of topological interlocking in forming robust composite materials. In this system, the biaxial expansion of the soft matrix by the functional skeleton had a large effect on toughness (energy dissipation), and the topological interlocking effect had a large effect on strength (stiffness and maximum force). Without requiring a strong interface, this method can create composite materials that exhibit excellent mechanical properties. Furthermore, since high interfacial strength is not a requirement, this opens the possibility for making a wide range of Macro-DN composites, with diverse materials combinations.

## Conclusion

In summary, we have demonstrated that incorporating functional skeletons into the Macro-DN composite design improves the resulting mechanical response, especially in the low stretch region. Enhanced stiffness and toughness seen in Macro-DN composites are now known to originate from two sources: (1) the incorporated auxetic or honeycomb structures that exhibit strongly negative or positive Poisson’s ratio mismatch (Δμ), increasing deformation of the matrix prior to skeleton rupture, and (2) the preferential, repetitive rupture of the skeleton within the composite prior to matrix fracture, based on the DN principle. The method introduced here improves upon previous Macro-DN designs that increased toughness solely through the fracture of a sacrificial network. This new method simultaneously increases the work performed by the matrix, by incorporating biaxial deformation. Even if the interfacial adhesion strength between the skeleton and the matrix is poor, it is possible to improve the mechanical properties of composite structures through topological interlocking. This strengthening mechanism can be widely used for diverse combinations of hard skeletons and may serve as a guideline for the design of high strength soft/hard composites in the future.

## Methods

### Materials

The precursor solution used for synthesizing a soft and stretchable silicone rubber as the matrix is a commercially available two-part kit: KE-1603-A, and KE-1603-B (Shin-Etsu Chemical) and was used as received without further purification. The model materials used to fabricate the hard skeleton were AR-M2 (model material) and AR-S1 (support material) and were purchased from Keyence Co. AR-M2 consists of an acrylate monomer, urethane–acrylate oligomer, and photoinitiator. AR-S1 consists of an acrylate monomer, polypropylene glycol, and photoinitiator.

### Skeleton fabrication

The plastic skeletons were designed by CAD software (Autodesk Inventor Professional 2019; https://www.autodesk.com/products/inventor/). Handles were designed on both ends of the geometric part. The geometry of the skeleton excluding the handles had a width of 24.5 mm, length of 60 mm, and thickness of 2 mm (Figure S1a). The functional geometries (auxetic, offset rectangle, and honeycomb) were designed by changing the angle (θ = 40°–140°) of the joints (Figure S1b). All interconnects have a width of 0.5 mm. A spacer was designed around the exterior of the skeleton to fix the skeleton in the middle of the matrix and maintain a total composite thickness of 4 mm and was removed prior to sample testing. These designed skeletons were 3D printed (AGILISTA-3000, Keyence Co.). After printing, the skeletons were washed in deionized water to remove the support material and dried.

### Composites fabrication

To make the composite, skeletons with an integrated spacer were placed on a glass plate. The silicone rubber precursor solution was prepared by mixing the two silicone components at a mix ratio of 1:1 in a vacuum mixer ((ARV-310, Thinky Co.), 2000 rpm, 30 kPa, 2 min). After mixing, the solution was poured into the mold and the glass plate was placed on a level table in air at 25 °C for 2 days to cure the silicone. After that, the sample was cut to specific dimensions (L_0_ × w_0_ × t_0_ = 60 × 27 × 4 mm^3^) using a laser cutter (PLS4.75, Universal Laser Systems). For the pristine silicone rubber (matrix), the samples were prepared with the same formulation and polymerization conditions as those of the composites for the mechanical tests in the absence of the plastic skeletons.

### Mechanical testing

#### Tensile test

Uniaxial tensile testing was performed on the composites, pristine silicone rubber (matrix), and the neat skeletons using a tensile-compressive tester (Instron 5965 type universal testing system). All samples were stretched along the length direction at an extension rate of 100 mm/min at room temperature. Stretch ratio, λ, is defined as *L*/*L*_0_, where *L*_0_ and *L* are the length of the sample before and during elongation, respectively. All stretching experiments were recorded visually with a video camera (Panasonic VX985M).

#### Cycle test

Cyclic loading/unloading tensile tests were performed on the composites, pristine matrix, and neat skeletons using a tensile-compressive tester (Instron 5965 type universal testing system). All samples were stretched to a stretch ratio of λ = 1.083 at a velocity of 100 mm/min. Then, samples were returned to the initial displacement immediately at the same velocity as stretching. This process was repeated 5 times for the samples.

#### Circular polarized imaging system

To observe the stress distribution during deformation and fracture of the samples, a homemade circular polarizing imaging system was combined with tensile testing. A white lamp and a video camera were set in front of and behind the sample, respectively. Two pieces of circular polarizer films were fixed, respectively, on the white lamp and video camera. The video camera recorded the shape and isochromatic images of the samples during stretching. This simple method allows us to qualitatively visualize the stress distribution of the samples during deformation.

## Supplementary Information

Supplementary Information 1.

Supplementary Video 1.

Supplementary Video 2.
