# Supplementary material for: Improving the strength and toughness of macroscale double networks by exploiting Poisson’s ratio mismatch
Source: Sci Rep. 2021 Jun 24;11:13280. doi: 10.1038/s41598-021-92773-0 (PMC8225664; doi:10.1038/s41598-021-92773-0)
Supplement: Supplementary file 1 — Supplementary Information 1. [file 41598_2021_92773_MOESM1_ESM.docx]

SUPPORTING INFORMATION

**Improving the Strength and Toughness of Macroscale Double Networks by Exploiting Poisson’s Ratio Mismatch**

*Tsuyoshi Okumura, Riku Takahashi^†^, Katsumi Hagita, Daniel R. King^*^, Jian Ping Gong^*^*

Mr. T. Okumura, Dr. R. Takahashi
Graduate School of Life Science, Hokkaido University, Sapporo, 001-0021, Japan.

Dr. K. Hagita
Department of Applied Physics, National Defense Academy, 1-10-20 Hashirimizu, Yokosuka 239-8686, Japan

Dr. D. R. King, Prof. J. P. Gong
Faculty of Advanced Life Science, Hokkaido University, Sapporo, 001-0021, Japan.

Prof. J. P. Gong
Institute for Chemical Reaction Design and Discovery (WPI-ICReDD), Hokkaido University, Sapporo, 001-0021, Japan.

*Corresponding Authors: [dking@sci.hokudai.ac.jp](mailto:dking@sci.hokudai.ac.jp); [gong@sci.hokudai.ac.jp](mailto:gong@sci.hokudai.ac.jp)

^†^Present Address: NTT Basic Research Laboratories, NTT Corporation, 3-1 Morinosato-Wakamiya, Atsugi, Kanagawa, 243-0198, Japan.

# Supporting information 1. Mechanical properties of component materials by uniaxial tensile testing.

**Table S1.** Mechanical properties of the component materials measured by uniaxial tensile testing. These values were measured using dumbbells (gauge region: l_0_×w_0_×t_0_ = 12×2×0.5 mm^3^) based on Japanese Industrial Standard. Data are the average of n = 3 measurements.

| Component | Young’s modulus  E (MPa) | Fracture Stress  σ (MPa) | Fracture Strain  ε | Work of Fracture  W (MJ m^-3^) |
| --- | --- | --- | --- | --- |
| Skeleton | 1017 ± 11.6 | 53.25 ± 1.88 | 0.078 ± 0.003 | 2.75 ± 0.26 |
| Matrix | 0.66 ± 0.02 | 4.27 ± 0.37 | 7.052 ± 0.541 | 15.99 ± 2.82 |

# Supporting information 2. Dimensions of the skeleton and composites.

The geometry of skeletons and their composites are shown in **Figure S1** and **S2**. Three types of skeleton geometries were fabricated. **Figure S1** shows a schematic illustration of the skeleton with auxetic structure (40°), as an example. The dimensions shown in **Figure S1** and **S2** are in mm, which were kept constant throughout this paper.

1) In **Figure S1a**, each end of the skeleton consists of a large rigid handle. These handles provide enough stiffness for the grips of the tensile tester, and ensures that the gauge length region undergoes deformation, not the regions gripped by the tensile tester.

2) The skeleton interconnects were fabricated with a constant width of 0.5 mm (**Figure S1b**) and a thickness of 2 mm. Three types, auxetic, offset rectangle, and honeycomb, with a total of 5 specific geometries were fabricated. These geometries were controlled by changing the internal angle (θ = 40° - 140°) (**Figure S1c**). The internal width of each cell was fixed at 5.5 mm. The length of each longitudinal segment (denoted as *L* in **Figure S1**) changes depending on the internal angle: 40°, 10 mm; 60°, 8.8 mm; 90°, 7.6 mm; 120°, 5.3 mm; 140°, 2.9 mm. A row with three cells and a row with four cells were designed respectively. Therefore, the skeleton has four rows with three cells and three rows with four cells alternating. Half-finished rows of four cells were added at both ends to unify the length of the skeleton without handle.

3) In **Figure S2a**, a schematic is presented of the skeleton in **Figure S1** embedded in silicone elastomer. Two cross-sectional images are shown corresponding with the colored planes in **Figure S2a**. **Figure S2b** is a cross-sectional image of the plane highlighted in red, containing the row of three cells. **Figure S2c** is a cross-sectional image of the plane highlighted green, containing the row of four cells. The sample is embedded on all sides by silicone rubber.

Figure S1. Dimensions of the skeleton with auxetic structure (40°). All dimensions are listed in mm. (a) Schematic of the skeleton. (b) Section of the red plane, the specific geometry by internal angle. (c) Schematic illustrations of the skeleton geometries utilized and their respective internal angles.

Figure S2. Schematics of the fabricated composite. All dimensions are listed in mm. (a) Schematic of the skeleton embedded in elastomer. (b) Cross-section of the red plane, containing three cells. (c) Cross-section of the green plane, containing four cells. The dark blue phase represents the skeleton while the light blue represents the matrix.

# Supporting information 3. Measurement of Poisson’s Ratio.

Figure S3. Optical images of the neat skeletons and their composites used for measuring the planar Poisson’s ratio. The measurements were performed at a stretch ratio λ=1.02 from the relation $\mu=-\frac{{(w-w_{0})}/{w_{0}}}{{(L-L_{0})}/{L_{0}}}$, using the data shown in the figures. w was the central width of the skeletons. (a) An auxetic skeleton. (b) A composite with auxetic skeleton. (c) A honeycomb skeleton. (d) A composite with honeycomb skeleton.

# Supporting information 4. Tensile tests performed with circular polarizing imaging system.

See **Video S1**.

# Supporting information 5. Mechanical properties of composites and pristine matrix by uniaxial tensile testing.

**Table S2.** Mechanical properties of the composites and pristine matrix by uniaxial tensile testing and volume fraction of the skeletons in the Macro-DN composite. Data are average of at least n = 3 trials of different samples prepared by the same run.

| Component | Structure | Angle,  θ (°) | Poisson’s ratio difference, Δμ | Poisson’s ratio,  μ | Initial Stiffness, κ (kN/m) | Yield Force, F_y_ (N) | Yield Stretch ratio,  λ_y_ | Work of Extension, W (mJ)  (λ=1~1.65) | Volume fraction,  φ_skeleton_ |
| --- | --- | --- | --- | --- | --- | --- | --- | --- | --- |
| Composite | Auxetic | 40 | -2.73 ± 0.13 | -0.90 ± 0.35 | 21.74 ± 0.59 | 68.52 ± 1.50 | 1.112 ± 0.006 | 2232 ± 63 | 0.130 |
| Composite | Auxetic | 60 | -1.63 ± 0.25 | -0.50 ± 0.21 | 13.21 ± 0.47 | 41.69 ± 1.84 | 1.121 ± 0.005 | 1730 ± 95 | 0.093 |
| Composite | Offset Rectangle | 90 | 0.26 ± 0.09 | 0.61 ± 0.09 | 8.96 ± 0.54 | 32.18 ± 2.05 | 1.174 ± 0.003 | 1338 ± 68 | 0.076 |
| Composite | Honeycomb | 120 | 2.43 ± 0.23 | 1.80 ± 0.25 | 9.05 ± 0.36 | 22.41 ± 0.85 | 1.054 ± 0.011 | 1245 ± 29 | 0.072 |
| Composite | Honeycomb | 140 | 3.56 ± 0.40 | 2.73 ± 0.13 | 15.26 ± 0.41 | 38.47 ± 1.85 | 1.043 ± 0.002 | 1526 ± 72 | 0.080 |
| Matrix | - | - | - | 0.48 ± 0.01 | 1.69 ± 0.02 | - | - | 733 ± 14 | - |

# Supporting information 6. Poisson’s ratio of neat skeletons and Macro-DN composites.

Figure S4. Experimental Poisson’s ratios, μ, of the neat skeletons (open symbol) and Macro-DN composites (filled symbol) measured at stretch ratio ofλ= 1.02. The numerical average of the skeleton and matrix for each angle (average value = $\frac{\mu_{skeleton}+\mu_{matrix}}{2}$) are shown as solid black symbols. The experimental Poisson’s ratios of the composites closely match the numerical average of the skeleton and matrix. The symbols represent the average of at least n = 3 trials and error bars represent the standard deviation.

# Supporting information 7. Yield stretch ratio of neat skeletons and composites.

Figure S5. Yield stretch ratio of the neat skeletons (open symbols) and Macro-DN composites (filled symbols) as a function of skeleton Poisson’s ratio. Triangles represent auxetic samples, and hexagons represent honeycomb samples. The colors and shapes of the symbols correspond with the internal angles in **Figure S4**. The symbols represent the average of at least n = 3 trials and error bars represent the standard deviation.

# Supporting information 8. Cycle test performed with circular polarizing imaging system.

See **Video S2**.
